# Physical activity preferences of individuals diagnosed with schizophrenia or bipolar disorder

**DOI:** 10.1186/s13104-016-2151-y

**Published:** 2016-07-12

**Authors:** Mehala Subramaniapillai, Kelly Arbour-Nicitopoulos, Markus Duncan, Roger S. McIntyre, Rodrigo B. Mansur, Gary Remington, Guy Faulkner

**Affiliations:** Faculty of Kinesiology and Physical Education, University of Toronto, 55 Harbord Street, Toronto, ON M5S2W6 Canada; School of Kinesiology, University of British Columbia, 2146 Health Sciences Mall, Room 4606, Vancouver, BC V6T 1Z3 Canada; Mood Disorders Psychopharmacology Unit, Toronto Western Hospital, University Health Network, 399 Bathurst Street, MP 9-325, Toronto, ON M5T 2S8 Canada; Schizophrenia Program Centre for Addiction and Mental Health, 250 College Street, Toronto, ON M5T 1R8 Canada

**Keywords:** Physical activity, Preferences, Severe mental illness, Health perceptions, Walking

## Abstract

**Background:**

Individuals with a severe mental illness (SMI) are at least two times more likely to suffer from metabolic co-morbidities, leading to excessive and premature deaths. In spite of the many physical and mental health benefits of physical activity (PA), individuals with SMI are less physically active and more sedentary than the general population. One key component towards increasing the acceptability, adoption, and long-term adherence to PA is to understand, tailor and incorporate the PA preferences of individuals. Therefore, the objective of this study was to determine if there are differences in PA preferences among individuals diagnosed with different psychiatric disorders, in particular schizophrenia or bipolar disorder (BD), and to identify PA design features that participants would prefer.

**Methods:**

Participants with schizophrenia (n = 113) or BD (n = 60) completed a survey assessing their PA preferences.

**Results:**

There were no statistical between-group differences on any preferred PA program design feature between those diagnosed with schizophrenia or BD. As such, participants with either diagnosis were collapsed into one group in order to report PA preferences. Walking (59.5 %) at moderate intensity (61.3 %) was the most popular activity and participants were receptive to using self-monitoring tools (59.0 %). Participants were also interested in incorporating strength and resistance training (58.5 %) into their PA program and preferred some level of regular contact with a fitness specialist (66.0 %).

**Conclusions:**

These findings can be used to tailor a physical activity intervention for adults with schizophrenia or BD. Since participants with schizophrenia or BD do not differ in PA program preferences, the preferred features may have broad applicability for individuals with any SMI.

## Background

Individuals with a severe mental illness (SMI), such as schizophrenia or bipolar disorder (BD), live 25–30 years less than the general population [1, 2]. Mortality studies indicate that the excess and premature deaths in SMI are largely a consequence of natural causes (e.g. cardiovascular disease, diabetes mellitus) rather than unnatural causes (e.g. suicide) [3, 4].

Furthermore, individuals with a SMI are at least two times more likely to suffer from metabolic co-morbidities, in particular abdominal obesity and type 2 diabetes, than the general population [5–10]. Individuals with schizophrenia are up to four times more likely to be overweight and obese than their otherwise healthy peers [11–13], which places them at an increased risk of cardiovascular disease [14, 15] and premature death. Cardiovascular disease accounts for 12–49 % of all-cause mortality in patients with schizophrenia [16] and 35–40 % in persons with BD [17], compared to only 20 % in the general population [18]. In addition to these individual-level health costs, there are significant societal health care costs associated with treating these secondary complications in persons with SMI [19].

There are several possible reasons for the increased incidence of cardio-metabolic disorders and associated chronic health conditions among persons with SMI. First, commonly used antipsychotic medications often lead to weight gain as a result of slowed metabolism, consumption of foods with high caloric content, and reduced resting energy expenditure [10, 20]. In particular, clozapine and olanzapine have been shown to be associated with the greatest weight gain among its users [1], as these antipsychotics have been shown to inhibit appetite suppression, leading to increased high caloric food intake [21, 22]. Second, poor lifestyle behaviours, such as consuming unhealthy diets, smoking, and physical inactivity further contribute to the progression of these diseases [9]. Individuals with SMI are also more sedentary then their healthy peers, and this behavior is itself associated with negative health outcomes, such as increased inflammatory markers [e.g. c-reactive protein (CRP)] [23]. Furthermore, simply having a diagnosis of a SMI is a risk factor for having cardio-metabolic disorders [24]. It is possible that because of the focus on the psychiatric diagnosis and management, individuals with a SMI as well as their health care providers may have neglected to understand or prioritize care for physical health and wellbeing [25]. Furthermore, individuals with SMI are often of low socioeconomic status and have poor access to healthcare [26]. Given what we now know, this issue needs to be addressed as aggressively and with the same urgency as their primary psychiatric diagnosis [27].

Within a broader, comprehensive plan to address these physical health concerns, the promotion of regular physical activity (PA) is critical [28]. There is extensive evidence supporting the benefits of regular PA, particularly in reducing the risk of metabolic diseases and the burden associated with being overweight or obese. Importantly, regular PA reduces the risk of cardiovascular disease through improved cardiovascular and respiratory function [29]. Furthermore, individuals who are active but obese have a reduced risk for all-cause and cardiovascular mortality compared to individuals who have a normal BMI but are physically inactive [30]. Therefore, regular PA incurs physical health benefits to the individual independent of weight loss. In addition, PA has been shown to promote better mental health. Specifically, it has been shown to alleviate depressive symptoms, particularly among those who are diagnosed with a SMI [31–33]. Therefore, PA may actually play a dual role in supporting better health outcomes in this vulnerable population.

In spite of the many physical and mental health benefits that PA has to offer, individuals with a SMI are less physically active [34–36] and more sedentary than the general population [37]. In a recent study that examined PA levels of individuals with BD, none of the 60 participants examined were meeting the national PA guidelines of 150 min of moderate-to-vigorous intensity PA per week and that, on average, participants spent 78 % of their waking time in sedentary activities [38]. Similar results were found among individuals with schizophrenia (n = 46), where they spent over 80 % of their waking time in sedentary behaviour, and achieved only 18.6 min of moderate-to-vigorous intensity PA per day [39]. Together, these findings point to the need to develop effective programs for promoting regular PA among individuals with SMI. One key component towards achieving this goal is to understand and incorporate the PA preferences of individuals with SMI into the PA program in order to increase the acceptability, adoption, and long-term adherence to PA. In line with “patient-centered care” frameworks [40], matching treatment approaches to patient preferences may enhance adherence to that treatment. In this case, if the most effective dose of physical activity is the one that individuals enjoy and can sustain [41], then matching physical activity options to preferences is recommended.

Previous research examining PA preferences among persons with SMI is limited. Three studies were conducted among psychiatric patients [42–44], and a third targeted individuals who self-reported non-specific psychological distress during the previous 4 weeks [45]. All studies reported that, in general, participants preferred low-to-moderate intensity PA that could be implemented within or outside the home, where walking was the most preferred type of PA. One gap within this literature is whether PA preferences differ among individuals with different types of SMI. This knowledge would be useful to a variety of stakeholders, such as health care providers, as a way to understand how certain features of PA programs (e.g., intensity, duration and type of PA) can be better tailored to meet the PA needs and interests within this diverse population. In addition, previous research among this population, particularly schizophrenia, has demonstrated the importance of social support [46, 47]. Although these studies exploring PA preferences reported whether or not participants wanted to receive support from a fitness instructor or health care professional, there was no detailed examination of what the specific role of this individual would be and the extent of their involvement. This type of information has practical implications for determining the components of a successful PA program.

Given these aforementioned limitations, the purpose of this study was twofold: (a) to determine whether individuals with schizophrenia differ from individuals with BD as to the preferred features of PA programs, and (b) to summarize the PA design preferences of these individuals, including the nature and extent of the involvement by a fitness specialist.

## Methods

### Participants

Participants with schizophrenia or BD were invited to take part in this study. All participants diagnosed with schizophrenia in the research registry (n > 100) at the Centre for Addiction and Mental Health in Toronto, Canada were approached to take part in this study and the Mini International Neuropsychiatric Interview (MINI; [48]) was used to confirm their diagnoses. All participants with physician-confirmed BD visiting the Mood Disorders Psychopharmacology Clinic (about 10 patients/week) at the Toronto Western Hospital in Toronto, Canada from August 2014 to March 2015 were asked to complete this questionnaire after an appointment with their physician.

### Ethics approval

This study was approved by the Research Ethics Boards at the University of Toronto (#29489), Centre for Addiction and Mental Health (#099/2013-01) and the University Health Network (#13-6799). Informed and voluntary consent was obtained from all participants.

### Measures

All participants completed a survey, responding to questions about their physical activity preferences. The following demographic information was collected: age, sex, ethnicity, employment status, weight and height, minutes of moderate-to-vigorous intensity PA completed during the previous week using the international physical activity questionnaire short form [49], and the self-characterization of regular PA using a stages of change algorithm [50, 51].

The questionnaire itself was divided into two sections. The first section surveyed participants on their personal PA preferences (e.g. type of activity, duration of sessions, intensity) and how they preferred a PA program to be structured (e.g. overall duration and when they wanted to start the program). Participants were also asked if they wanted to be active with others who share the same diagnosis as this aspect may serve as a way to improve self-efficacy and act as a social support mechanism [52]. A question relating to the use of PA monitoring devices, such as a pedometer, was also posed, as these tools may serve to help participants track their progress as well as serve as a motivating factor [53, 54]. Strength and resistance training is critical for maintaining optimal bone and muscle health [55]. However, this is often a neglected component in many PA regimens. Therefore, a question about incorporating this type of training was also included.

Although it has been previously reported that individuals with a SMI want support from a fitness instructor or a health care provider to be active [44], the nature and extent of the support that participants want has not been previously elucidated and therefore, difficult to translate into practice. In order to address this limitation and understand the level and kind of support that participants would like to receive from a fitness specialist, the second part of the questionnaire asked participants to rank three different types of PA programs. These different program scenarios were originally presented to individuals with substance use disorders to gather insight as to their PA preferences [56] (see Table 1 for a full description of these scenarios). In general, Program A would provide no additional support beyond an initial consultation with a fitness specialist. In Program B, participants are provided with brief regular contact with a fitness specialist and given additional resources (e.g., fitness monitoring tools and gym memberships), although participants continue to determine the specific parameters of how they will be active as in Program A. In contrast, in Program C, participants follow a predetermined program once a week for 12 weeks that includes an aerobic PA session and an education component. The participant and fitness specialist plan together the PA schedule that the participant will independently follow for the following week.

**Table 1:** Three different types of physical activity programs that participants ranked from most preferred to least preferred [54]

Program	Description
*Program A* Do it yourself	1. Participate in a brief session with a fitness specialist to discuss the benefits of physical activity and how to get started
	2. How, when and where you are physically active will be solely determined by you
*Program B* Do it youself with professional guidance	1. Be given fitness tools to help you monitor your progress (e.g., heart rate monitors, pedometers) OR a membership to a community fitness center where you could engage in physical activity whenever it is convenient for you
	2. Exercise guidance and consultation will be provided by an fitness specialist. This will consist of an initial session and continued contact with the fitness specialist through brief phone calls and/or occasional visits to a fitness facility
	3. How, when, and where you exercise will be determined by you, with guidance from the fitness specialist
*Program C* Relying on professional supervision	1. Attend a fitness facility at the same predetermined day and time each week for 12 weeks. At these weekly sessions, you will: (a) Engage in aerobic exercise under the direction of a fitness specialist, and (b) participate in a group to learn about and discuss the benefits of physical activity
	2. How, when, and where you are physically active during the week will be determined through discussions with the fitness specialist

### Data analyses

Chi square analyses were conducted to determine if there were any between-group differences on the measured demographic variables or PA preference parameters among those diagnosed with schizophrenia compared to those diagnosed with BD. Descriptive statistics (i.e. percentages) were conducted to determine the most preferred PA program design features for future interventions among all participants, noting any particular differences based on gender, age, specific psychiatric diagnoses, and whether participants self-classified themselves as active or inactive. A median split was used to examine the preferences of younger adults (age ≤39, n = 89) and older adults (age >39, n = 84).

## Results

### Participant characteristics

Demographic and anthropometric information are presented in Table 2. One hundred and thirteen adults with schizophrenia (schizophrenia [66.4 %], schizoaffective [30.7 %], psychosis NOS [2.1 %]) and 60 participants with BD (BD I [36.7 %], BD II [60.0 %], BD NOS [3.3 %]) took part in this study. All participants were outpatients from the community and both groups of participants were comparable in age (schizophrenia M = 40.0 years, SD = 11.7; BD: M = 41.6 years, SD = 12.7). There were more men in the schizophrenia group (62.8 %) than in the BD group (48.0 %), although this difference was not significant. Participants with schizophrenia self-reported, on average, 185.4 min of moderate-to-vigorous PA (MVPA) (SD = 188.5) during the previous week, although 85.8 % participants were not meeting PA guidelines set forth by the World Health Organization of 150 min of moderate-intensity or 75 min of vigorous-intensity exercise per week [57]. Participants with BD self-reported, on average, 246.8 min of MVPA (SD = 228.0), although 41.7 % of participants were not meeting recommended guidelines. Furthermore, 68.2 % of participants with schizophrenia or 61.6 % of participants with BD self-characterized themselves as physically inactive (i.e., not currently achieving 150 min/week of MVPA) based on the stage of change algorithm. On average, participants with schizophrenia were obese (BMI: M = 31.5 kg/m^2^, SD = 8.4 kg/m^2^; 54.0 %) whereas participants with BD were in the range between being overweight and obese (BMI: M = 28.2 kg/m^2^, SD = 6.4 kg/m^2^; 26.7 % [overweight], 35.0 % [obese]; Table 2). Overweight (BMI = 25.0–29.9 kg/m^2^) and obesity (BMI ≥30.0 kg/m^2^) were classified based on the World Health Organization’s BMI classification [58]. This difference in mean BMI between both groups was statistically significant (*t* [168] = 2.66, *p* = 0.01). No other significant between-group differences were found on the remaining demographic variables (all *p*s > 0.05).

**Table 2:** Demographic and anthropometric information for individuals with schizophrenia (SZ) or bipolar disorder (BD)

Demographics	SZ (N = 113)	BD (N = 60)
	N (%)	N (%)
Male:female (N)	68:45	29:31
Mean Age (SD), in years	40.20 (11.5)	41.58 (12.7)
*BMI (kg/m* ^*2*^ *)*		
Mean ± SD	31.5 (8.4)	28.16 (6.4)
Normal weight (≤24.9)	20 (17.7)	19 (31.7)
Overweight (25.0–29.9	32 (28.3)	16 (26.7)
Obese (≥30.0)	61 (54.0)	21 (35.0)
*Ethnicity*		
White	67 (59.3)	51 (85.0)
African	19 (16.8)	1 (1.7)
Asian	9 (8.0)	2 (3.3)
South Asian	7 (6.2)	0
Biracial/multiracial/other	11 (9.7)	5 (8.3)
*Employment*		
Not employed	64 (56.6)	27 (45.0)
Mean MVPA (SD), min/week	185.35 (188.5) (n = 94)	246.81 (228.0) (n = 58)
*Stages of change (physical activity)*		
Precontemplation	2 (1.8)	2 (3.3)
Contemplation	20 (17.7)	15 (25.0)
Preparation	55 (48.7)	20 (33.3)
Action	18 (15.9)	7 (11.7)
Maintenance	18 (15.9)	16 (26.7)

Values are counts and percentages unless otherwise specified

*MVPA* moderate-to-vigorous physical activity

### PA preferences

Chi square tests for independence indicated no significant association between the specific mental health diagnosis (schizophrenia vs. BD) and PA preferences. Since there were no between-group differences on any of the PA preference items, the results from these descriptive analyses were collapsed and are presented in Table 3.

**Table 3:** Preferred physical activity program parameters for individuals with schizophrenia or bipolar disorder

*Session parameters*	
Where	Outdoors (52.0 %)
	Gym/fitness center (48.6 %)
	Home (37.0 %)
Duration	One long bout (e.g. 30 min) (62.4 %)
Intensity	Moderate (61.3 %)
	High (27.7 %)
	Low (22.5 %)
Types of activities	Walking (59.5 %)
	Swimming (37.5 %)
	Yoga/stretching (37.5 %)
	Gym/fitness club (37.0 %)
	Cycling (31.2 %)
Requires individualization	Time of day morning (23.7 %), afternoons (33.5 %), evenings (24.9 %), no preference (26.0 %)
	With whom (others with or without the diagnosis), no preference (50.9 %)
	Women’s only class ( 40.8 %)
*Program parameters*	
When to start	Now (60.7 %), next 12 weeks (15.0 %)
Program length	12-week program (51.4 %), 6-week program (21.4 %)
*Other*	
Use of pedometers?	Yes (68.2 %)
Add strength/resistance training?	Yes (67.6 %)

#### Overall receptivity for PA programs

Participants, in general, were receptive towards participating in a PA program, particularly if the program was designed for individuals with their specific psychiatric diagnosis (59.5 %). Among participants who self-reported being physically inactive based on the stages of change model, this preference was slightly higher (63.2 %). The majority of participants preferred to have both men and women in the class (38.7 %) or had no preference regarding class gender (39.3 %). Forty-one percent of women preferred women’s only classes. Over half (60.7 %) of all participants reported that they would like to enrol in a PA program immediately, particularly those who reported being inactive (55.3 %). Fifteen percent  of participants wanted to start a PA program within the next 12 weeks.

The most popular program option from the scenarios provided was Program B (48.6 %; ‘do it yourself with professional guidance’), even among those participants who were inactive (48.2 %). However, there was a sizable number of participants who also preferred Program A (22.0  %) and Program C (27.7 %).

#### Preferred PA program characteristics

The majority of participants preferred a PA program that was of longer (i.e., 12 weeks [51.4 %]) versus shorter (i.e., 3 weeks [16.2 %], 6 weeks [21.4 %], 9 weeks [12.7 %]) duration. Participants preferred to be active for a set period of time (e.g., one bout of 30 min; 62.4 %) as opposed to breaking up their PA throughout the day in shorter bouts (e.g., 10 min; 15.6 %). The majority of participants preferred to be active at a moderate intensity (61.3 %), as opposed to low (22.5 %) or high (27.7 %) intensities. Younger adults (33.7 %) preferred high intensity exercise to older adults (21.4 %), and older adults (28.6 %) preferred low intensity PA to younger adults (16.9 %). Highlighting the need for some flexibility in any potential programming, there was no consensus as to the best time of day when the PA program should be offered (mornings [23.7 %], afternoons [33.5 %], evenings [24.9 %] and no preference [26 %]).

Walking (59.5 %) was the most popular type of PA chosen and participants were receptive to using pedometers (68.2 %) to keep track of their daily steps. Swimming (37.5 %), yoga/stretching (37.5 %), being active at a fitness center (37.0 %) and cycling (31.2 %) were similarly endorsed by participants. However, yoga/stretching was more popular among women (52.6 %) than men (25.8 %) and cycling was more popular among men (38.1 %) than women (22.4 %). The majority of all participants (67.6 %), particularly those diagnosed with BD (78.3 %) and those who were inactive (64.9 %), also wanted to incorporate strength/resistance training within their PA program, regardless of the particular type of aerobic PA they chose. Compared to younger adults, older adults preferred low impact PA, such as walking (71.4 vs. 48.3 %) and swimming (45.2 vs. 30.0 %). Younger adults preferred activities of higher intensities when compared to older adults, such as running (22.5 vs. 8.3 %) and team sports (23.6 vs. 13.1 %). With regards to location, the majority of participants wanted to be active primarily outdoors (52.0 %), at a gym or a fitness center (48.6 %), or at home (37.0  %). Some participants also wanted their PA program to take place in a clinical/hospital setting (11.0 %) and others (18.5 %) had no particular preference as to where they wanted to be active.

## Discussion

In sum, this examination of the PA preferences among individuals diagnosed with schizophrenia or BD revealed no between-group differences on any particular PA program design feature or intervention parameter that was assessed. Notably, these preferences are similar to the PA preferences commonly reported among the general population—a low-to-moderate intensity activity either outdoors, at a fitness center or at home, with walking being the most popular activity [42, 45, 59, 60]. This is an encouraging finding for health care providers because a PA program with the features noted above can be made available to individuals living with a SMI, without having to consider personalizing the program to a particular diagnosis. This greatly improves the feasibility and broader applicability of PA program design across a wide range of health care and community settings.

The results from this study largely support the findings described by Ussher et al. [44], Carpiniello et al. [42] and Fraser et al. [43]. Walking was the most preferred form of PA and the desire for social support, particularly from a fitness instructor or a health care professional, by participants to be active was consistent across all four studies. The similarity in the latter finding is not surprising considering that lack of social support is highly correlated with physical inactivity in this population [34, 61]. Furthermore, the use of low-to-moderate PA and social support have played a critical role in the success of lifestyle and weight management interventions among individuals with SMI, such as the STRIDE [62], In SHAPE [63] and ACHIEVE [64] programs. However, differences emerged with regards to where participants wanted to be active. In contrast to the preference to do home-based PA reported by Ussher et al. [44], outdoor and fitness-center based PA were popular, with home-based PA preferred by a small number of participants, in this present study as well as the study by Carpiniello and colleagues [42].

This study has investigated a number of variables that have not been examined previously, particularly the PA preferences among individuals with different types of psychiatric diagnoses, specifically schizophrenia or BD. This is also the first study to examine the kind of support that participants want to receive from a fitness specialist. By presenting participants with three possible scenarios of different levels of involvement by a fitness specialist, it provided participants with a detailed description of the role of the fitness specialist for their easy comprehension. The use of scenarios will also facilitate the communication of this information to stakeholders. Other novel preference parameters examined in this present study are the receptivity towards using self-monitoring tools and the incorporation of strength and resistance training. These additional findings will further assist health care providers to design an appealing PA program for individuals with a SMI.

The interpretation of, and inferences that may be arrived at from our results, are affected by several methodological limitations. First, this is a cross-sectional examination of preferences that were self-reported by participants. Although only three broad PA programs were provided to participants to rank in this study, there may be other PA program designs that may be of greater appeal to participants. Interviews and focus groups with individuals with SMI would be beneficial to further understand how best to design a suitable PA program for these clinical populations. Intervention work would then be needed to confirm whether programs developed to match these preferences engage participants both in the short and long term. Additionally, preferences may change as individuals begin a PA program (e.g. based on their ability). Therefore, although findings are informative for developing a PA intervention for inactive individuals, some flexibility will be required in modifying the program based on a participants’ physical activity level and changing interests.

Finally, while identifying preferences for characteristics of a PA program is important for intervention design, greater consideration is needed in helping individuals with a SMI overcome the significant barriers, such as amotivation [42, 65, 66] to starting such a program. Other perceived barriers to PA reported by psychiatric patients include tiredness, the illness itself, and lack of enjoyment of PA [42]. In a recent meta-analysis examining various exercise interventions for physical and mental health benefits among individuals with schizophrenia, none of the 20 included studies employed a theoretical framework to systematically change behaviour [67]. Theory-based PA interventions in the general adult population have been shown to be more effective at increasing PA than atheoretical interventions [68]. Previous research has explored and tested the reliability of two theoretical models [health action process approach (HAPA) and self-determination theory (SDT)] to determine the most salient variables that can predict PA among individuals with schizophrenia at different stages of behaviour change [69, 70]. A theoretical framework, such as these, will be needed to inform intervention work helping individuals initiate and sustain participation in a PA program.

## Conclusions

In general, participants with schizophrenia or bipolar disorder report wanting to participate in a PA program and more than half of the participants wanted to enrol in a PA program immediately. These findings are in line with qualitative research demonstrating that many individuals with a SMI want support in leading healthier lives [71, 72]. Although there was some level of interest in a variety of activities, walking was, by far, the most popular option. Therefore, a walking program at moderate intensity that takes place outside would likely be an appealing option to many participants. Since participants were receptive to the use of pedometers, it is recommended that the PA intervention incorporate this tool to allow participants to self-monitor their progress over time. Personal assessment and monitoring may also serve as a motivational tool to help participants stay active [73]. A walking program may also be an effective way to build confidence and self-efficacy to consider other forms of PA in the future [74]. Since half of the participants wanted a PA program that was offered specifically to individuals with their diagnosis, a walking program may be a feasible addition for mental health centers and hospitals that see these patients regularly or have an inpatient unit. Furthermore, it is recommended that strength and resistance training also be made available to participants in addition to the walking program as there is high receptivity towards this type of exercise. In fact, a combined aerobic and strength training program has been previously shown to be well received by inpatients at a mental health facility [75]. This type of exercise has also been shown to be effective at improving cognitive, physical and psychiatric symptom-related outcomes among individuals with SMI [76, 77]. These considerations are now informing our own intervention work in these populations.

In sum, incorporating the PA preferences of individuals with SMI in future PA programs is an important basis for increasing PA participation needed to promote physical and mental health, while also contributing to weight management efforts [64, 78]. Preferences do not appear to vary by diagnosis, which should stream line efforts to develop appealing PA programs. However, any program incorporating these preferences needs to be part of a theoretically driven approach that addresses broader barriers to participation in this population.

## Abbreviations

BD: bipolar disorder
BD: NOS bipolar disorder not otherwise specified
BMI: body mass index
CRP: C-reactive protein
M: mean
HAPA: health action process approach
MINI: mini international neuropsychiatric interview
MVPA: moderate-to-vigorous physical activity
PA: physical activity
SD: standard deviation
SDT: self-determination theory
SMI: severe mental illness
